# Collecting sociodemographic data in primary care: qualitative interviews in community health centres

**DOI:** 10.3399/BJGPO.2024.0095

**Published:** 2025-03-26

**Authors:** Rachel Thelen, Sara Bhatti, Jennifer Rayner, Agnes Grudniewicz

**Affiliations:** 1 Telfer School of Management, University of Ottawa, Ottawa, Canada; 2 Alliance for Healthier Communities, Toronto, Canada

**Keywords:** primary health care, general practice, sociodemographic data collection, qualitative research

## Abstract

**Background:**

Many primary care organisations do not routinely collect sociodemographic data (SDD), such as race, sex, or income, despite the importance of these data in addressing health disparities.

**Aim:**

To understand the experiences of primary care providers and staff in collecting SDD.

**Design & setting:**

A qualitative interview study with 33 primary care and interprofessional team members from eight Ontario community health centres (CHCs).

**Method:**

Semi-structured virtual interviews were conducted between July and August 2021. The interviews were recorded and transcribed verbatim. Content analysis of the transcripts was undertaken.

**Results:**

Participants reported using both formal methods of SDD collection, and informal methods of SDD collection that were more organic, varied, and conducted over time. Participants discussed sometimes feeling uncomfortable collecting SDD formally, as well as associated burden and limited resources to support collection. Client–provider rapport was noted as facilitating data collection and participants suggested more training, streamlined data collection, and better communication about purpose and use of data.

**Conclusion:**

SDD can be collected informally or formally, but there are limitations to informally collected data and barriers to the adoption of formal processes.

## How this fits in

The literature indicates that sociodemographic data (SDD) collection is low in primary care settings and, when done, and should be flexible and complement clinical workflows. We studied community health centres (CHCs), which collected SDD with varying success. Our study results add reports from a variety of health professionals on the advantages of informal SDD collection methods (trust and rapport) but disadvantages for data capture at the organisational level, and reported provider, staff, and client discomfort with formal collection methods. The findings complement other recent work in the field (for example, building on understanding of 'comfort’ as an important theme during SDD collection) and can inform future data collection efforts.

## Introduction

Social determinants (for example, income, housing, and employment) influence health and drive disparities.^1–5^ Even in jurisdictions with universal access to healthcare, patients face disparities in access and outcomes.^4,6^ Access to high quality primary care is associated with more equitable health outcomes;^7^ however, strategies must also be adopted within primary care settings to combat inequity.^4^ SDD collection can improve health disparities,^4,5^ but routine collection and use in primary care is low.^8,9^

This study explores SDD collection in CHCs in Ontario, Canada. CHCs are primary care organisations that offer comprehensive healthcare services delivered by interprofessional teams, and are mandated to serve marginalised communities and promote equity.^10,11^ They are governed by community-elected boards, are non-profit, and offer salary-based remuneration to all providers. CHCs have long collected SDD to address barriers related to social determinants; however, some centres do not meet target collection rates (80% of clients).

The literature on SDD collection and use in healthcare settings explores collection methods; acceptance from providers,^12^ and patients and the public;^9,13,14^ feasibility;^15^ and barriers.^12^ There remains a lack of knowledge of facilitators to quality data collection,^12,16^ and a broader representation of health settings^17,18^ and professionals is needed. Since SDD collection is important to identify and combat disparities, we explored interdisciplinary provider and staff experiences collecting SDD in CHCs, and what factors facilitate collection and overcoming challenges. The aim of this was to learn what does and does not work well in the primary care context.

## Method

The research was done in partnership with Ontario’s CHC membership organisation, the Alliance for Healthier Communities (Alliance). An action-oriented approach^19^ was taken to explore a problem identified by CHCs with SDD collection rates. An invitation email was sent from the Alliance to executive directors (EDs) at 74 CHCs in Ontario, Canada. We recruited CHCs on a first-come, first-served basis within regions, to facilitate geographic representation. Eight CHCs were recruited. EDs and/or administrative staff from each of these CHCs helped recruit participants from their centre by sharing study invitations (*n* = 33).

### Data collection

Two researchers (RT and SB) conducted 30 minute semi-structured interviews by phone or video conference with interdisciplinary team members in July and August 2021. Our researchers are experienced in conducting qualitative and mixed-method research in primary care (AG, JR, SB) and have graduate-level qualitative research training. The interview guide (Supplementary Appendix 1) was informed by a review of literature on SDD collection in healthcare settings. It covered the following points: SDD collection goals; perceived impact and outcomes of collection (for example, on roles, workflow, clients); communication with clients during collection (we refer to patients as 'clients' because this is the language CHC participants used); collection processes; challenges and supports needed; facilitation strategies; and preferences and recommendations.

### Data analysis

Interviews were recorded and transcribed verbatim (detailed notes were taken for three participants who did not consent to recording). Qualitative content analysis^20^ was conducted using NVivo (version 12) software.

First, we prepared to focus on the descriptive, manifest content (for example, people involved, timing of collection, challenges, facilitators) that was close to the data and concrete (that is, not distant and abstract content such as sighs).^20,21^ We gained familiarity across the dataset by reading transcripts.^20^ Next, to organise data we combined inductive and deductive approaches to coding^20,21^ based on the literature, interview guide, and emerging topics. Two analysts (SB, RT) independently coded three identical transcripts, then met to compare inter-rater agreement early in analysis; code definitions and disagreements were discussed and resolved. Remaining transcripts were split to be coded independently using the same strategy. During and after coding, the analysts discussed and established categories describing similarities, differences, and hierarchies; to maintain quality and relevance, we ensured categories fit between the data and research questions.^20,22^ We explored relationships among categories to discover themes. While categories are specific and distinct, themes are more general and connect similarities among separate categories.^21,22^ We analysed all transcripts and concluded analysis when no new codes, categories, or themes emerged.^20^ Finally, to report results, we organised findings into three themes.

## Results

Thirty-three interdisciplinary team members (for example, family physicians, nurses, dieticians, administrative staff) from eight CHCs participated in semi-structured, individual interviews (see Table 1). Most participants were employed full-time (*n* = 26, 79%) and female (*n* = 29, 88%), and had been employed at their CHC for 7.9 years on average. We present our findings in three categories, including reported SDD collection and input methods, challenges and facilitators of SDD collection, and perspectives on collecting SDD.

**Table 1.** Study sample (*n* = 33)

Demographic	*n*	(%)	Years
Full-time position	26	79%	N/A
Female	29	88%	N/A
Mean years employed at CHC	33	N/A	7.87
**Roles**			
**Primary care providers**	**16**	**48%**	
Nurse practitioner (NP)	6	38%^a^	
Family physician	5	31%	
Registered practical nurse (RPN)	4	25%	
Registered nurse (RN)	1	6%	
**Interprofessional team members**	**12**	**36%**	
Team lead	4	33%	
Social worker	2	17%	
Dietician	2	17%	
Health promoter	1	8%	
Respiratory therapist	1	8%	
Client access worker	1	8%	
Supervisor	1	8%	
**Management and other staff**	**5**	**15%**	
Director	2	40%	
Administrative assistant	1	20%	
Medical receptionist	1	20%	
Medical secretary	1	20%	
**Total participants**	**33**	**100%**	

a Percentages below main role category are the percentage per role category, not per cent of all participants. CHC = community health centre

### Reported SDD collection and input methods

**Figure 1.**
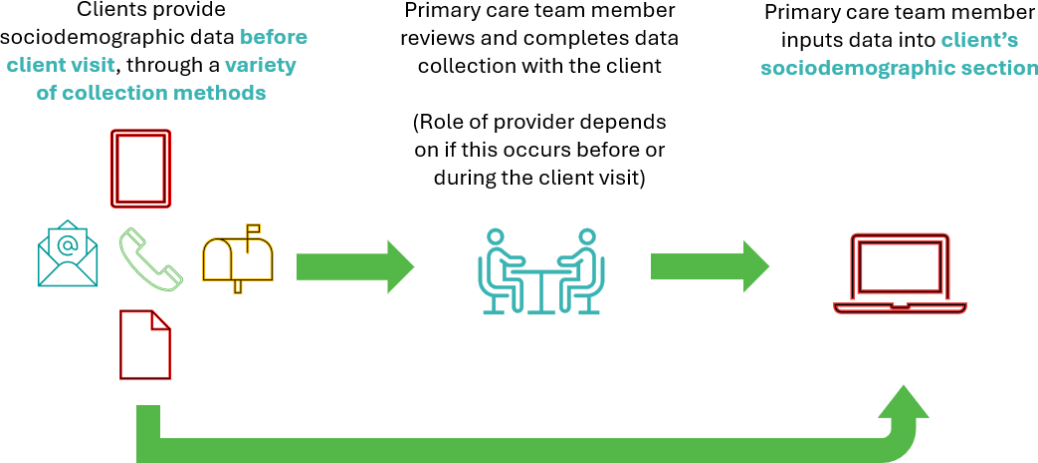
Formal collection methods

Participants reported using both formal and informal SDD collection methods. Formal methods (see Figure 1) were described as intentional, where clients provided SDD through a form and the data were inputted into a section in the client’s electronic medical record (EMR) for use by the CHC. Data collected included education level, age, income, ethnic and racial identity, gender, languages, household composition, health and accessibility (ongoing conditions), newcomer status, and wellbeing (sense of community belonging, self-rated health). Some teams customised these forms. Centres usually designated team members to assist clients and input collected data. Sometimes providers reviewed forms for completeness during the client visit.

**Figure 2.**
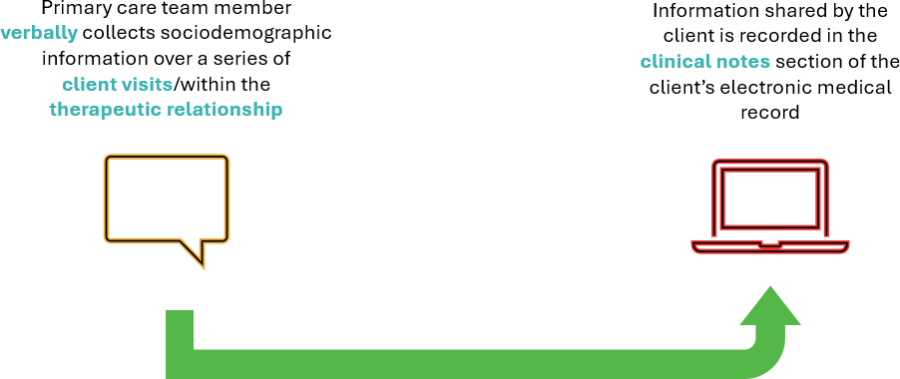
Informal collection methods

Informal methods varied in their breadth of questions, timing, input, and staff involved. They were described as organic collection of sociodemographic information verbally during client visits over time as the client–provider rapport developed (see Figure 2). Questions were subject to provider discretion. Sometimes this informal method occurred instead of formal collection. Data collected are entered in a free-text clinical note, making it challenging to retrieve for centre use. A nurse practitioner explained the challenge with data entered into a clinical note:

*'I'll do a short blurb … it doesn't actually get captured into the* [SDD template] *unless somebody goes back and* [inputs it].' (P4, CHC 5)

### Challenges and facilitators of SDD collection

#### Discomfort

Many participants said they, other team members, or clients sometimes become uncomfortable during collection. A client access worker shared the following comment:

*'I hate that question* [about income]*, and I understand why we ask it. I do. But that’s one of them that I'm like, “Wow. You're really getting personal."'* (P24, CHC 2)

Some participants attributed incomplete collection to discomfort. They also discussed how questions about race, sexual orientation, and gender can force people into a box with which they do not identify. A medical secretary said:

'[Clients] *really don’t want to give their income or their sexual orientation. Some people laugh and make jokes … Other people are just really offended that we’re even asking*.' (P28, CHC 1)

#### Burden

Participants from some centres reported SDD collection requirements to receive funding for programmes (for example, motherhood programme) or client access to services (for example, local food bank) in addition to collecting their own SDD for centre use. This resulted in redundant collection because while the data were similar, collection and input efforts were separate. A nurse practitioner said:

*'We have numerous emails coming every day, saying, "You need to be collecting this, you need to be collecting that*" *… We're constantly bombarded.'* (P4, CHC 5)

Formal collection is redundant if team members already talked with clients about sociodemographics verbally. Formal collection processes were also described as burdensome for clients. A medical secretary said:

*'It is a lot of extra work, like a lot, and* [clients] *don't want to* [complete forms]*, so it’s difficult. It’s not an easy thing to collect.'* (P28, CHC 1)

#### Limited resources

Participants explained that SDD collection and input takes extra time that teams do not have. Sometimes dedicated personnel were not available to collect and input SDD. A registered practical nurse said:

*'It’s very difficult when you have all these things that you have to do with the client and have to add a whole bunch of questions on top of it*.' (P32, CHC 8)

#### COVID-19 pandemic challenges

Most participants reported that the COVID-19 pandemic stopped or slowed collection. Other participants said some clients could not access forms at home. A medical receptionist said:

*'*[SDD collection] *usually falls on the medical receptionists … But it’s hard because we're short-staffed … we try to get* [SDD forms to clients] *through email or mail. And it’s getting* [forms] *back that’s now our concern.'* (P17, CHC 6)

#### Facilitators

Client–provider rapport and having SDD collection within a job description or scope of practice were reported as facilitators to SDD collection.

Participants at many centres said they wanted training on how to explain the purpose of collection to clients to foster comfort during the process. A physician described how they built comfort and rapport:

*'It’s not necessarily always in the first encounter that you're trying to capture* [SDD]*. It’s that growing relationship where people are feeling comfortable, and you learn a little bit more about where they're from and who they are*.' (P11, CHC 3)

Participants explained that it would be less burdensome if similar but separate collection efforts were streamlined. They also suggested that only the most pertinent items be collected (for example, key areas relevant to the centre’s targets). Participants also said SDD collection and input was facilitated when many team members were involved (rather a single person or role). Some participants suggested dedicated collection post-COVID-19 pandemic, but could not imagine having capacity.

### Perspectives on collecting SDD

#### SDD is useful for supporting client care

Most participants acknowledged the importance and usefulness of SDD within CHCs. A social worker shared that SDD provides:

*'… a sense of who we're serving and what our community looks like,* [and] *what our community needs might particularly be, which helps us to advocate for some funding, services, programs.'* (P27, CHC 1)

A nurse practitioner said:

*'Even just for prescribing medications, does a person have drug coverage? Are they in a lower socioeconomic status? … That’s kind of the way I use a lot of that information*.' (P3, CHC 7)

#### Formal SDD collection is not always appropriate

Some participants did not think formal SDD collection is necessary for all clients (for example, those using recreational services but not medical care). Others said formal collection can interrupt client care. Some SDD items themselves were seen as inappropriate. A nurse practitioner said:

*'We come from a small conservative community, you don't ask people who they have sex with. Although our front staff is great and they're trained and they know why they need to do it, in theory. It’s hard.'* (P13, CHC 4)

Many providers mentioned that formal collection is not useful when they already know longstanding clients and prefer to ask clients on a need-to-know basis.

#### Communication about SDD collection

Participants from some CHCs said they are frequently reminded to collect SDD. However, participants from only three of eight centres reported receiving updates about collection rates. Many participants described wanting to know how formal collection is going to motivate and affirm collection efforts. A registered practical nurse stated:

*'It would be nice to know exactly what the goal is behind* [collection] *… They tell us that it’s for programming, but beyond that, I don't really know.'* (P31, CHC 8)

#### Scope of practice

When formal collection was viewed beyond a provider’s scope of practice, it sometimes created tension. One nurse practitioner said they '*want to concentrate more on patient care than* [administrative] *work*' (P9, CHC 3), and a physician said, collection '*takes away from clinical work … In medicine, the administrative burden is becoming greater and greater. So, adding to that in any way is a real negative*' (P11, CHC 3).

Team members reported frequently discussing sociodemographic information within the therapeutic relationship (for example, between family physician and client, between dietician and client). These participants value sociodemographic information, but not the formal data per se, as they discussed SDD with clients during the client visit regardless of if they had access to formal data. A nurse practitioner said:

*'I don’t typically open the intake form. I don’t. I have a framework by which I ask my* [own] *questions … to get a comprehensive opening picture*.' (P25, CHC 1)

## Discussion

### Summary

We discovered formal and informal methods to SDD collection. We learnt that informal methods were preferred as they were facilitated by client–provider rapport and aligned with provider scope of practice. We found that participants were mostly willing to collect and input SDD because they valued it, although some team members only want to be involved informally. Formal methods of SDD collection made some participants uncomfortable. However, while preferred, informal methods do not systematically capture comprehensive SDD for centre needs. Participants wanted more communication about collection progress and use.

### Strengths and limitations

Our large sample size of participants from various primary care and interprofessional roles is a strength, although we did not include clients. Transferability of our results may be limited by the equity-focused nature of CHCs and the Canadian context. Furthermore, the SDD collection processes are understood through individual in-depth interviews but observation of clinical workflow in collecting SDD was not conducted.

### Comparison with existing literature

Our findings are consistent with previous findings that healthcare workers value SDD^4,12^ and want to address social determinants,^23^ but do not always see collection as necessary or appropriate^12^ (for example, it is seen as premature, private, and irrelevant). Previous literature highlights the importance of flexibility, to align with teams’ unique needs and workflows,^17,18^ while recognising the need for some standardisation.^12,18^ Challenges with workflow alignment are reported in many studies,^4,12,18,24^ and our participants also reported limited time to collect and input SDD.

Our study aligns with others that report discomfort with SDD collection and fear of negative consequences (for example, discrimination, negative client reaction) from patients and providers,^9,12,14,16,18,23,24^ and different comfort levels with different SDD items.^14,16,25^ The literature shows that training can increase provider and staff comfort,^12,16,23,24^ which our participants recommended. Browne *et al* reported that cultivating relationships is essential to supporting equity-oriented services because it takes time to develop trust and see health outcomes improve for marginalised populations.^4^ SDD can be collected to improve health disparities, but rapport needs to be developed with the most marginalised patients and over time within the therapeutic relationship.

No participants in our study expressed legal concerns for collecting SDD as found in another study as a challenge in the collection of race, ethnicity, and primary language data in health settings;^24^ however, some participants expressed the need to justify or explain collection. Other studies have also found that when patients understand the link between sociodemographic factors (for example, income) and health, they are more comfortable sharing that information in healthcare settings.^9^

### Implications for research and practice

Primary care organisations looking to collect SDD need to consider the advantages and disadvantages of both formal and informal approaches to collection. To improve likelihood of success, SDD collection should fit within workflows and be included in the scope or job description of specific staff. Training on how to build rapport and ask sociodemographic questions is recommended as well as providing staff with information on the purposes of collection, collection rates and targets, and use of the data. Future research should explore how to integrate formal and informal methods to utilise rapport, consider scope of practice, and ensure breadth and quality of SDD collection in meeting target collection rates.

In conclusion, CHCs have many years of experience collecting and using SDD data to support equity.^26^ This qualitative study discovered two SDD collection approaches in CHCs and explored challenges, facilitators, and the perspectives of collectors. Primary care settings should consider which approach or combination of approaches aligns with their needs and communicate the purpose of SDD collection to their teams.

## Data

The dataset relied on in this article is available from the corresponding author on reasonable request.
